# Necroptosis in tumorigenesis, activation of anti-tumor immunity, and cancer therapy

**DOI:** 10.18632/oncotarget.10548

**Published:** 2016-07-12

**Authors:** Mao-Bin Meng, Huan-Huan Wang, Yao-Li Cui, Zhi-Qiang Wu, Yang-Yang Shi, Nicholas G. Zaorsky, Lei Deng, Zhi-Yong Yuan, You Lu, Ping Wang

**Affiliations:** ^1^ Department of Radiation Oncology, Tianjin's Clinical Research Center for Cancer and Key Laboratory of Cancer Prevention and Therapy, Tianjin Medical University Cancer Institute & Hospital, National Clinical Research Center for Cancer, Tianjin, China; ^2^ Department of Lymphoma, Tianjin's Clinical Research Center for Cancer and Key Laboratory of Cancer Prevention and Therapy, Tianjin Medical University Cancer Institute & Hospital, National Clinical Research Center for Cancer, Tianjin, China; ^3^ Stanford University School of Medicine, Stanford, CA, United States of America; ^4^ Department of Radiation Oncology, Fox Chase Cancer Center, Philadelphia, PA, United States of America; ^5^ Department of Thoracic Cancer and Huaxi Student Society of Oncology Research, West China Hospital, West China School of Medicine, Sichuan University, Sichuan Province, China

**Keywords:** necroptosis, tumorigenesis, anti-tumor immune, cancer therapy

## Abstract

While the mechanisms underlying apoptosis and autophagy have been well characterized over recent decades, another regulated cell death event, necroptosis, remains poorly understood. Elucidating the signaling networks involved in the regulation of necroptosis may allow this form of regulated cell death to be exploited for diagnosis and treatment of cancer, and will contribute to the understanding of the complex tumor microenvironment. In this review, we have summarized the mechanisms and regulation of necroptosis, the converging and diverging features of necroptosis in tumorigenesis, activation of anti-tumor immunity, and cancer therapy, as well as attempts to exploit this newly gained knowledge to provide therapeutics for cancer.

## INTRODUCTION

In multicellular organisms, the balance between cell death, proliferation and differentiation is crucial for the maintenance of organ development, tissue homeostasis, and aging. For many years, the three basic types of cell death, type I, II, and III, could be distinguished according to morphological, enzymological, and functional criteria. Type I, apoptotic cell death, is defined by an ensemble of morphological features including chromatin condensation, nuclear fragmentation, cell shrinkage, plasma membrane blebbing, and the formation of an apoptotic bodies. Type II, autophagic cell death, is a process by which cells generate energy and metabolites by digesting organelles or macromolecules. Type III, necrosis, is characterized by a lack of stereotypical morphological changes, but does eventually result in the rounding of the cell, cytoplasmic swelling, and the rupture of the plasma membrane and the spilling of the intracellular contents [1–2].

The field of cell death research has rapidly developed, resulting in the recommendation of updated cell death classification criteria by the Nomenclature Committee on Cell Death [2]. For example, necrosis has for a long time been considered an accidental mode of cell death. However, it was recently recognized that a form of cell death morphologically classified as necrosis could also be regulated in a programmed manner *via* defined signal transduction pathways (termed necroptosis) [3–5]. Multiple lines of evidence indicate that necrosis can be a programmed event: (I) cell death with a necrotic appearance can contribute to embryonic development and adult tissue homeostasis; (II) necrotic cell death can be induced by ligands that bind to specific plasma membrane receptors, (III) necrosis can be regulated by genetic, epigenetic, and pharmacological factors [6]; (IV) the cellular disintegration phase of necrosis is characterized by an identical sequence of sub-cellular events, including oxidative burst, mitochondrial membrane hyperpolarization, lysosomal membrane permeabilization and plasma membrane permeabilization, although with different kinetics [7]; and (V) the inactivation of caspases causes a shift from apoptosis either to cell death morphologies with mixed necrotic and apoptotic features or to full-blown necrosis [8].

The molecular mechanisms involved in necroptosis have been intensively studied in recent years. In principle, a multitude of different stimuli can initiate necroptosis, comprising mainly of three phases of signal transduction, including an initiation and an execution phase associated with the loss of cell and organelle integrity. The execution necroptosis phase involves activation of specific death mediators, such as receptor-interacting protein kinases (RIPKs) and mixed-lineage kinase domain-like protein (MLKL) [9–10].

Accumulating evidence indicates that necroptosis is involved in the regulation of cancer [11–16]. It is widely accepted that evasion of cell death is one of the hallmarks of cancer [17–18]. Many lines of clinical and experimental evidence have demonstrated that defects in cancer cell death are the most frequent causes of therapeutic resistance, and thus exploring cancer cell death might inform development of strategies to overcome therapeutic resistance. Although the molecular mechanisms underlying necroptosis need to be further elucidated, it is becoming clear that further insights into the signaling networks involved in regulation of necroptosis will likely have important implications for the exploitation of this form of regulated cell death for the diagnosis or treatment of cancer in the complex tumor microenvironment. With these aims in mind, in this review, we summarize the role of necroptosis in tumorigenesis, activation of anti-tumor immunity, and cancer therapy.

## MECHANISMS AND REGULATION OF NECROPTOSIS

**Figure 1:**
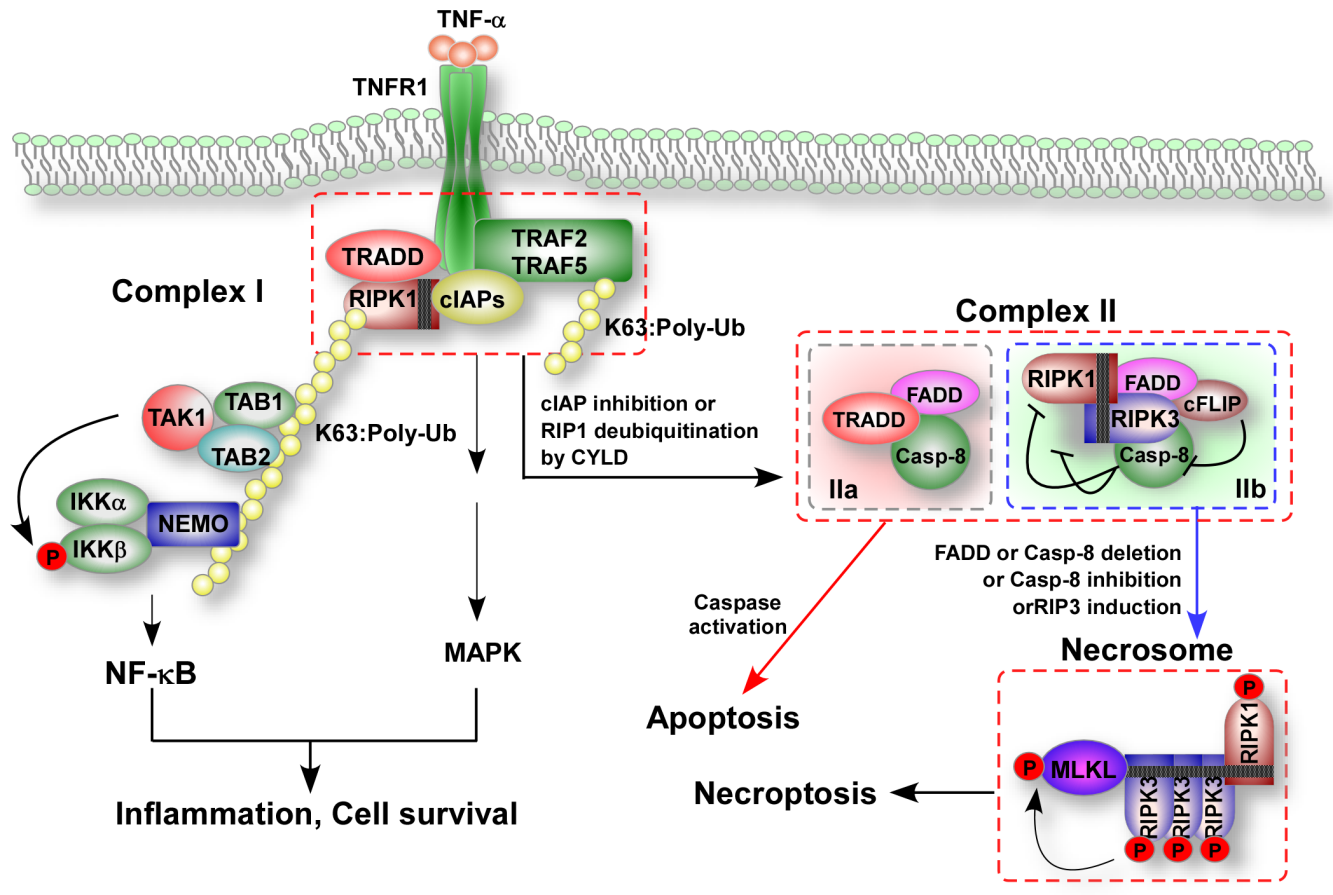
TNF-induced formation of apoptotic and necroptotic signaling complexes After ligand binds to the receptor, the intracellular tails of tumor necrosis factor receptor 1 (TNFR1) recruit multiple proteins to form the membrane-proximal supramolecular structure complex I including TNFR1 associated death domain protein (TRADD), receptor-interacting protein kinase-1 (RIPK1), cellular inhibitors of apoptosis (cIAPs), the E3 ubiquitin ligases TNF-receptor-associated factor 2 and 5 (TRAF2 and TRAF5). Lys63-linked polyubiquitination (K63-poly Ub) of RIPK1 in complex I mediated by cIAP ligases is crucial for the recruitment of nuclear factor-κB (NF-κB) essential modulator (NEMO), a regulatory subunit of IκB kinase (IKK) complex that in turn activates NF-κB and mitogen-activated protein kinases (MAPKs). Deubiquitination RIPK1 by cylindromatosis (CYLD) or inhibition of cIAP proteins promote the conversion of complex I to complex II and inhibits NF-κB activation. Complex II contains RIPK1, Fas-associated protein with death domain (FADD), caspase-8, cellular FLICE-inhibitory protein-L (cFLIP_L_), RIPK3 and TRADD. Caspase-8 becomes activated in complex II and initiates apoptosis, whereas cFLIP_L_ can prevent activation of caspase-8. In cells with high levels of RIPK3 expression, RIPK3 enters complex II *via* interaction with RIPK1 after stimulation. The RIPK3-containning complex is called complex IIb or the necrosome. In the presence of cFLIP_L_, caspase-8 is unable to initiate apoptosis but can cleave RIPK1 and RIPK3 and thus inhibits necroptosis. Depletion of FADD or caspase-8, inhibition of caspase-8 or induction of RIPK3 can free RIPK1-RIPK3 from inhibition and initiate necroptosis by mixed-lineage kinase domain-like protein (MLKL) of TNF-treated cells.

Considering the emerging significance of necroptosis in cancer, a better understanding of the molecular mechanisms underlying necroptotic signaling will likely have important implications for the development of novel methods to interfere with necroptosis in cancer. In principle, a multitude of different stimuli can initiate necroptotic cell death, which mainly comprises three phases of signal transduction, including an initiation and an execution phase, finally causing the loss of cell and organelle integrity and cell death (Figure 1).

### Initiation of necroptosis

Necroptosis can be elicited by a range of stimuli, from cytokines, viral infection, chemicals, or damage-associated molecular patterns (DAMPs), to several forms of physicochemical cellular stress [19]. Different necroptotic stimuli are recognized or sensed by specific receptors or sensors on the cell surface or cell interior. A range of receptor-sensor complexes can initiate the necroptotic response to different stimuli, although the nature of some of these complexes is presently unknown. We focus on the components of TNF-α signaling, because this signaling pathway is the most extensively studied inducer of necroptosis [9]. Under some conditions, TNF signaling occurs primarily through TNF receptor 1 (TNFR1), a potent inducer of induced cell death. However, early evidence demonstrated that TNF induces caspase-independent cell death by a mechanism involving activation of RIPK1 [3, 20–21]. Also important is the identification of necroptotic inhibitor necrostatin-1 (Nec-1) targeting RIPK1 that indicated that TNF-α induced necroptosis is a kinase-regulated process [16, 22]. In addition, RIPK3 was reported to be an essential regulator of TNF-α induced necroptosis [22–24], and TNF-α stimulation was reported to induce the formation of a necrosome in which RIPK3 is activated to interact with RIPK1 *via* the RIP homotypic interaction motif (RHIM) initiating necroptosis [23–25].

**Figure 2:**
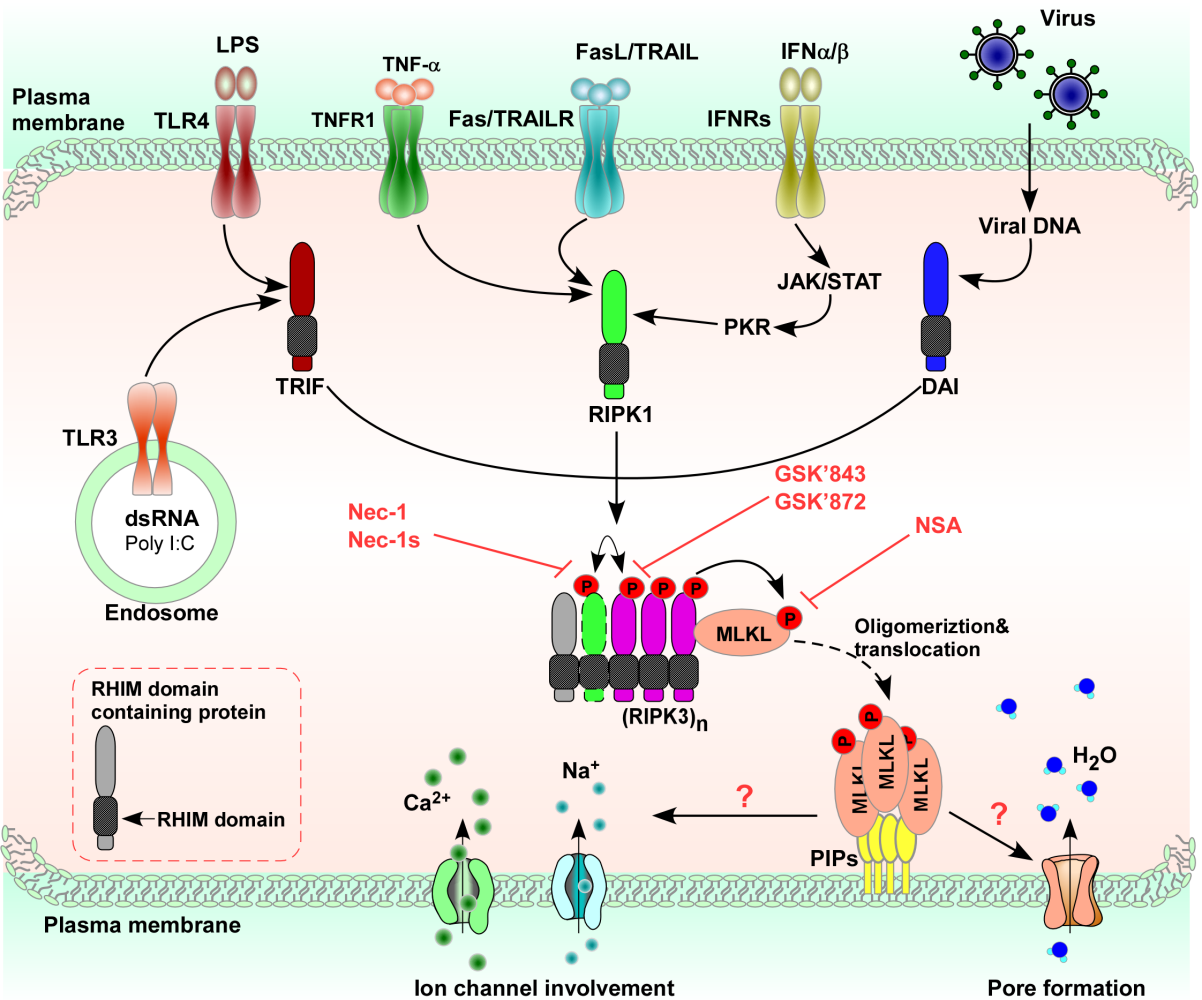
Necroptosis is induced by various stimuli Necroptotic stimuli such as TNF-α, FasL/TRAIL, IFN-α/IFN-β, pathogen-associated molecular patterns (LPS, poly (I:C)) *via* TLR activation or virus-mediated activation of DAI are recognized or sensed by specific receptors or sensors on the surface of or inside cells, and mediate the initiation of the necroptotic response to different stimuli, although the nature of some of these complexes is currently unknown. Formation of the necroptotic-signaling complex determines cell date. Different necroptotic complexes are found after ligation of different receptors or under genotoxic stress. When different necroptotic stimuli induced necroptosis, a complex called the necrosome is formed leading to RIPK3 oligomerization. MLKL could then modify ion influx, changing osmotic pressure, and/or forming a pore, thus causing plasma membrane rupture. In addition, necroptosis can be pharmacologically inhibited by Nec-1 (RIPK1 inhibitor), GSK- 843/872/840 (RIPK3 inhibitors), and NSA (MLKL inhibitor).

Besides TNF-α, there are five different stimulators of necroptosis. (I) Fas or tumor necrosis factor related apoptosis inducing ligand receptor (TRAILR): stimulation of Fas or TRAILR induces formation of the receptor-bound death inducing signaling complex that triggers caspase-8 mediated apoptosis independent of RIPK1. Under particular conditions, such as the absence of cellular inhibitors of apoptosis (cIAPs), which favor the recruitment of RIPK1 to Fas [26], and the formation of a cytosolic ripoptosome complex [27], which mediates necroptosis when caspase-8 is blocked. (II) Toll-like receptor-4 (TLR4) and Toll-like receptor-3 (TLR3): TLR4 or TLR3 stimulation triggers formation of the necrosome through the RHIM containing adapter Toll/IL-1 receptor domain containing adaptor protein inducing interferon (IFN)-β (TRIF), resulting in RIPK3 dependent necroptosis in which the role of RIPK1 depends on the cellular context. (III) double-stranded DNA viruses such as murine cytomegalovirus (MCMV): DNA dependent activator of IFN regulatory factors recognize viral double-stranded DNA and, through its RHIM domain, recruits RIPK3 to induce formation of the necrosome without RIPK1, triggering RIPK1 independent RIPK3 kinase activity dependent necroptosis. (IV) IFN-α and IFN-β: IFN-α and IFN-β induce necroptosis through their cognate receptors, interferon alpha receptors (IFNRs), leading to activation of the Janus kinase/Signal transducer and activator of transcription (JAK/STAT). In cells that were deficient in nuclear factor κ-light-chain-enhancer of activated B cells (NF-κB) signaling, IFN-γ promoted accumulation of mitochondrial reactive oxygen species (ROS) and eventual loss of mitochondrial membrane potential that ultimately leads to necroptosis; and (V) polyinosine-polycytidylic acid (Poly (I:C)): Poly (I:C)-TLR3 stimulation in the absence of zVAD-fmk induced TRIF mediated necroptosis (Figure 2).

### Execution of necroptosis

Efforts to further explore the execution of necroptosis include establishment of a robust cell-based model of necroptosis. By treating human colon cancer HT-29 cells with TNF-α, about 200,000 chemical compounds were screened for their ability to inhibit necroptosis, and MLKL was identified as a critical substrate of RIPK3 during the induction of necroptosis [28]. Meanwhile, the fact that MLKL pseudokinase is a substrate of RIPK3 required for necroptosis sheds light on the mechanisms involved in executing necroptotic cell death downstream of RIPK3 [29], as RHIM-dependent oligomerization and intramolecular autophosphorylation of RIPK3 results in the recruitment and phosphorylation of MLKL at Ser 345, Ser 347, and Thr349 [30–32]. Further studies demonstrated that the phosphorylation of MLKL at Ser 345 is not required for interaction between RIPK3 and MLKL in the necrosome, but is essential for MLKL translocation, accumulation in the plasma membrane, and consequent necroptosis [33]. Taken together, two non-exclusive models are proposed for the executioner mechanism of MLKL: one as a platform at the plasma membrane for the recruitment of Ca^2+^ or Na^+^ ion channels [34–35], and the other as a direct pore-forming complex that is recruited through binding of the amino-terminus of the 4-helical bundle domain of MLKL to negatively charged phosphatidylinositophosphates [36–38].

In addition, phosphoglycerate mutase 5 (PGAM5), a mitochondrial phosphoglyceratemutase, was identified to be another necrosome-associated protein that can regulate dynamic-related protein (Drp1) through its dephosphorylation [39–40]. It is noteworthy that the two variants of PGAM5 (PGAM5S and PGAM5L) have different functions during necroptosis, even though they are both required for necroptosis execution. After the necrosome core is formed, PGAM5L binds to the necrosome, unaffected by the MLKL inhibitor necrosulfonamide (NSA); however, NSA blocks PGAM5S. In addition, multiple leading cell death laboratories have recently reported that PGAM5 is dispensable for necroptosis [41–42]. Therefore, although many cellular events have been reported to act downstream of the necroptotic-signaling complex to execute necroptosis, the role of PGAM5 during necroptosis remains largely elusive and somewhat controversial. Further investigation will be required to clarify the importance of these executors of necroptosis.

### Mechanisms of necroptosis

After TNF-α binds to the receptor TNFR1, the intracellular tails of TNFR1 recruit multiple proteins to form the membrane associated protein Complex I, which contains TNFR1 associated death domain protein (TRADD), RIPK1 and the E3 ubiquitin ligases TNF-receptor-associated factor 2 and 5 (TRAF2 and TRAF5), cIAPs, and the linear ubiquitin chain assembly complex (LUBAC) [3, 43–44]. This complex provides a platform for the recruitment of downstream kinases and effector proteins to activate NF-κB and mitogen-activated protein kinase (MAPK) [45–51]. NF-κB and MAPK are believed to initiate a survival pathway because they induce expression of some genes encoding cytoprotective molecules [52].

Under some conditions, the intracellular tails of TNFR1 also induce apoptosis through cytosolic complex IIA (TRADD, Fas-associated protein with death domain (FADD), and caspase-8) and IIB (RIPK1, RIPK3, FADD, caspase-8, and cellular FLICE-inhibitory protein-L (cFLIP_L_)), and in particular conditions or cells, necroptosis can be executed by necrosomes including RIPK1, RIPK3, and MLKL [53]. Destabilization of complex I leads to the formation of a second cytosolic complex IIA, which induced apoptosis and inhibits NF-κB activation [54–55]. In conditions such as TNF stimulation in the inhibition of cIAPs [52] or deubiquitination of RIPK1 by cylindromatosis (CYLD) [56–58], a cytosolic IIB complex forms, precipitating apoptosis. In cells with high levels of RIPK3 and MLKL expression, or under the conditions when caspase-8 activity is reduced, blocked or absent; complex IIB may form the necrosome [59].

### Mechanisms determining the type of cell death

It is clear that programmed cell-death signaling pathways share some common components. For example, the same death stimuli can trigger different modes of cell death according to the molecular complexes present in the pathway. Different modes of cell death can also occur simultaneously or separately depending on the cellular circumstances.

Currently, the precise mechanisms that determine progression to necroptosis remain poorly understood. Numerous studies have suggested that expression of RIPK3 and MLKL correlates with sensitivity to necroptosis [24, 36, 60–64], however, a potential drawback of these studies is that RIPK3 and MLKL expression were compared between healthy and inflamed tissues. It is, therefore, difficult to conclude that RIPK3 and/or MLKL over-expression have primary causal functions.

Caspase-8 is the most crucial factor for preventing necroptosis. TNF-α induced signaling towards necroptosis is prevented by caspase-8, and it is reported to inhibit necroptosis by cleaving RIPK1 [65] and RIPK3 [66], as well as CYLD [67]. Currently, sensitization to necroptosis is achieved through a genetic defect compromising FADD-caspase-8 signaling and thus inhibiting apoptosis [68–71]; and inhibition of caspase-8 causes necroptosis [24], suggesting that whether a cell dies by apoptosis or necroptosis depends on caspase-8 activity.

In addition, ubiquitination or deubiquitination of RIPK1 can regulate the TNFR1 signaling pathway, driving necroptosis *in vitro* and *in vivo* [60, 68, 72]. In contrary, several brakes on RIPK1, affecting the presence or absence of RIPK1 and its post-translational modifications such as ubiquitylation and phosphorylation, can also inhibit necroptosis. Although ubiquitination has clearly emerged as a major regulatory mechanism, the substrates, exact linkage composition of the different chains, and the precise roles of the diverse related enzymes remain to be clarified [73–75].

## NECROPTOSIS IN TUMORIGENESIS

Necroptosis was recently reported to play a very important role in tumorigenesis as a backup cell death mechanism in cancer cells. Hitomi *et al*. [72] used a genome-wide screen using small interfering RNAs to delineate a cellular signaling network that regulated necroptosis and implicated two suppressor genes, CYLD and EDD1, and four Ras related proteins in regulation of necroptosis, which suggested a possible function of necroptosis in tumorigenesis.

Yang *et al*. [76] reported that the ratio of RIPK3-r, a truncated splice variant of RIPK3, to RIPK3 was significantly increased in colon and lung cancers relative to matched normal tissues, indicating that RIP3-r may be a major splice form associated with tumorigenesis. Furthermore, the RIPK3 gene is located on chromosome 14q11.2, a locus frequently altered in many cancers including nasopharyngeal carcinoma and T cell leukemia/lymphoma [77]. In non-Hodgkin lymphoma, polymorphisms in the RIPK3 gene were identified and found to correlate with increased risk of tumors [78].

Mutations in the CYLD gene in tumorigenic epidermal cells increase the aggressiveness of carcinomas, mainly by enhancing expression of angiogenic factors, thereby playing a key role in epidermal cancer malignancy [79–80]. In chronic lymphocytic leukemia cells (CLL), RIPK3 and CYLD were downregulated and lymphoid enhancer-binding factor 1 (LEF1) acts as a transcription repressor for CYLD [81]. Taken together, necroptosis may play a very important role in tumorigenesis [82].

## NECROPTOSIS BACKS UP TUMOR IMMUNE ACTIVATION

**Figure 3:**
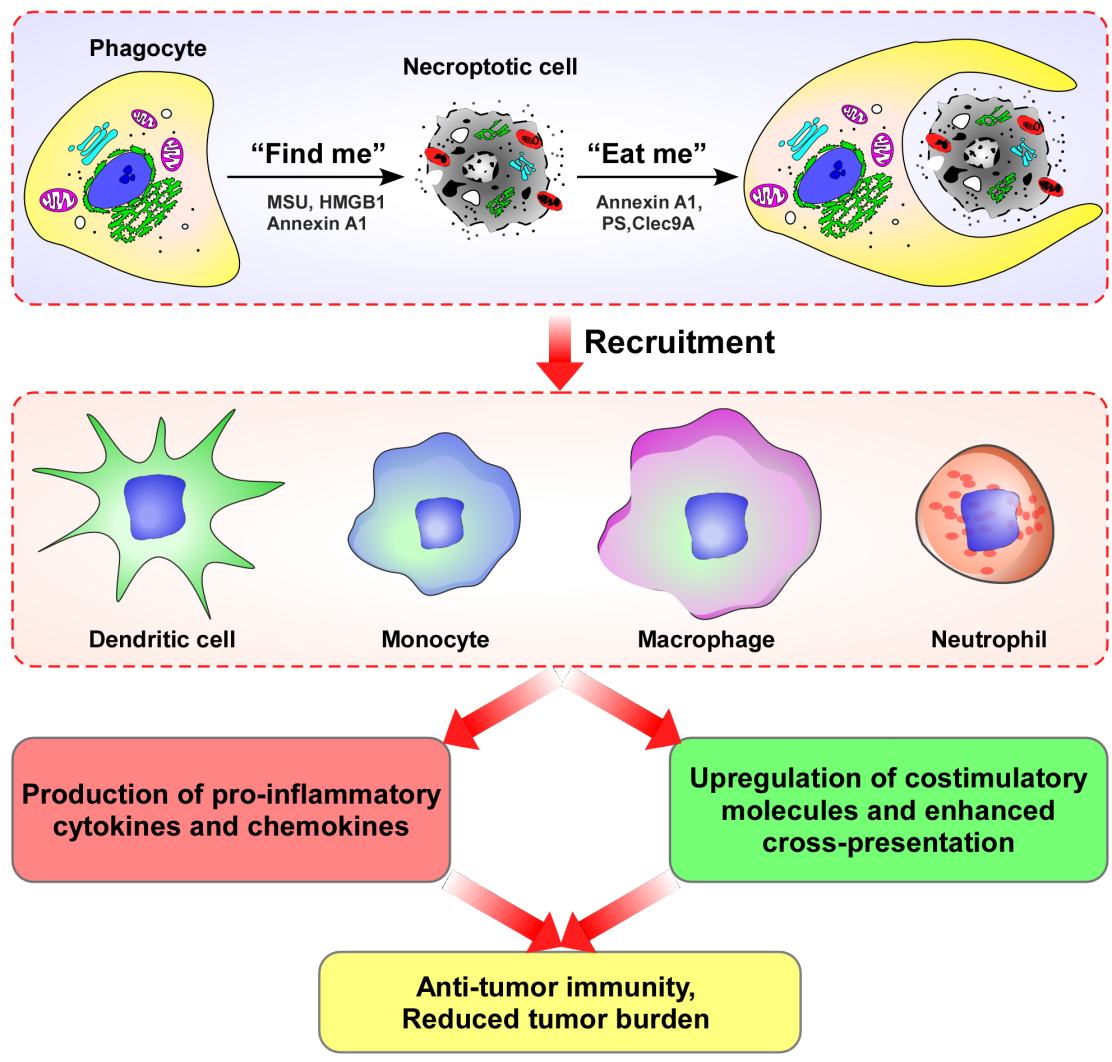
The concept that cell death may precede, trigger or amplify immunity has gained increasing attention Immune activation phagocytic cells (dendritic cells, macrophages, monocytes, and neutrophils) can identify and engulf necroptotic tumor cells, inducing production of pro-inflammatory cytokines and chemokines, up-regulation of stimulatory molecules and enhanced cross-presentation; and, eventually, triggering of adaptive immune response.

The idea that cell death may precede, trigger or amplify immunity has recently gained increasing attention. Similar to apoptotic cells, necroptotic tumor cells can induce anti-tumor immunity, necroptotic tumor cells can be cleared by innate immune phagocytic cells and engulfed by dendritic cells, macrophages, monocytes, and neutrophils, inducing release of pro-inflammatory cytokines and chemokines, upregulation of stimulatory molecules and enhanced cross-presentation, and eventual trigger of adaptive immune responses (Figure 3).

### Necroptotic cell clearance

Higher-level organisms have developed impressively efficient immune mechanisms to clear dying cells, for which the capability to distinguish dying from viable cells is crucial [83]. The sophisticated process of apoptotic cell removal involves several distinct phases, phagocyte recruitment, the engulfment of target cells, and the post-phagocytic response [84–86]. Phagocyte recruitment is accomplished by the release of soluble “find-me” signals from the dying cell [87–88]. Similar to apoptotic cells, the most likely candidates for necroptotic cell derived “find-me” signals are monosodium urate (MSU) crystals [89], high mobility group box 1 protein (HMGB1) [90], and annexin A1 [91–92]. When phagocytes reach the dying cells, they identify and recognize “eat-me” signals, which are exposed on the surface of the dying cell. Specific “eat-me” signals of necroptotic cells include annexin A1 [92–93], phosphatidylserine (PS) [94–95], and the C-type lectin Clec9A [96–98]. After dying cells are engulfed, phagocytic cells, including dendritic cells, macrophages, monocytes, and neutrophils can produce pro-inflammatory cytokines and chemokines, up-regulate stimulatory molecules and enhance cross-presentation, eventually triggering adaptive immune responses [99–100]. In addition, Martinez *et al* found that LC3-associated phagocytosis may play a key role in the clearance of dying cells and inflammation in the control of systemic lupus erythematosus [101]. Of course, further studies will be required to clarify the mechanisms of necroptotic cell clearance.

### Effect of necroptosis on pro-inflammatory cytokine production

Necroptosis is thought to directly trigger inflammation through a massive release of DAMPs including HMGB1, interleukin family cytokines, adenosine triphosphate (ATP), and so on, which are released by necrotic cells [102]. However, necrotic cells can inhibit inflammatory reactions under some conditions [103–104]. For example, macrophages can engulf necroptotic L929 cells without producing inflammatory cytokines [98], highlighting an unexpected complexity in the immune response to necrotic cells. Theoretically, acute release of DAMPs could enhance the pro-inflammatory effect of necroptosis; however, the role of specific DAMPs in necroptosis induced in inflammation has not been functionally validated. While DAMPs appear to be involved in necroptosis-induced inflammation, their role awaits *in vivo* experimental validation.

### Necroptotic cell induction of anti-tumor immune response

Dying cells induce DAMPs and initiate adaptive immunity by providing both antigens and inflammatory stimuli for DCs, which in turn activate CD8^+^ T cells through antigen cross-priming [105–106]. For example, IFN-α/β predominantly affects macrophages, DCs, and natural killer (NK) cells; inducing their activation and/or maturation, upregulating of chemokine expression, enhancing antigen-presentation and cross-presentation by DCs and a robustly augmenting induction of adaptive immune responses [107].

Anticancer agents induce a type of cell stress and death that is immunogenic; directly killing cancer cells, and using dead cells to provoke innate and adaptive immunity to attack other cancer cells [108]. For example, some studies demonstrated that stimulation of cervical cancer cells with PolyI:C, induced necroptotic cell death, then necroptotic cancer cells released IL-1α, which was required for the strong activation of DCs to produce IL-12, a cytokine critical for anti-tumor immune responses [109–110]. Kang *et al*. [111] implicated RIPK3-PGAM5-Drp1 signaling in NKT cell activation, and further suggested that RIPK3-PGAM5 signaling may mediate crosstalk between mitochondrial function and immune signaling. Aaes *et al*. reported that necroptotic cancer cells release DAMP and promote DC maturation, cross priming of cytotoxic T cells, and production of IFN-γ in response to tumor antigen stimulation [112]. Recently, using the model of cervical cancer, Smola demonstrated that PolyI:C driven immunogenicity strictly depends on the necroptosis regulator RIPK3 in neoplastic cells, suggesting RIPK3 as a novel predictive marker for personalization of cancer immunotherapy [113].

Although necrosis may induce effective immune responses, out of control necrosis could potentially cause irreversible tissue damage [114]. Additionally, the immune inflammatory cells recruited by the inflammatory factors released by necrotic cells may promote angiogenesis and cancer cell proliferation [115]. By tolerating some degree of necrotic cell death, invasive and metastatic tumors may gain growth-stimulating factors from the recruited tumor-promoting inflammatory cells [6]. Therefore, role of necroptosis of cancer and anti-tumor immune responses may be difficult to elucidate.

### Necroptosis in T-cell expansion

Several reports indicate that necroptosis regulates the antigen-induced proliferation of T cells required for peripheral T cell homeostasis and T cell survival in response to activation stimuli [116]. For example, mice lacking caspase-8, or expressing a dominantly interfering form of FADD unable to recruit caspase-8 in T cells, exhibited impaired T cell homeostasis and diminished peripheral T cell numbers [117]. Impaired expansion of T cells after Fyn-T cell receptor (TCR) activation was observed in T cells deficient in caspase-8 [118–119] or FADD [120], as well as in FADDdd-expressing T cells [121–122]. Furthermore, blockade of RIPK1 through Nec-1 restores the expansion defect in caspase-8 deficient and FADDdd-expressing T cells [123–124], as does the genetic knockdown of RIPK1 in FADD deficient mice [72]. Similarly, loss of RIPK3 can rescue defective T cell proliferation of caspase-8−/− or FADDdd mice [125–126], indicating that necroptotic signaling in T cells is regulated by caspase-8.

Osborn *et al*. [120] demonstrated that necroptosis occurs during the late stage of normal T-cell proliferation, and that this process is greatly amplified in FADD-deficient T cells. Furthermore, in a thymidine kinase transfected tumor mouse model, the necrosis induced by a vascular target agent ZD6126, impaired the function of tumor-specific CD8+ T cells and abrogated a robust tumor specific immune response [127]. Cho *et al*. demonstrated that Nec-1 can inhibit RIPK1 dependent and independent necroptosis, and that a high dose of Nec-1 will inhibit other signal transduction pathways such as that for T cell receptor activation [128]. In addition, regulatory T cells (Tregs) are potent immune regulators and play a key role in maintaining peripheral tolerance. The contribution of two forms of programmed cell death, apoptosis and RIPK dependent necroptosis, to Treg-mediated suppression were evaluated, and it was found that Tregs can't mediate suppress apoptosis or necroptosis *via* programmed cell death pathways [129]. Therefore, the capacity of necroptosis to trigger tumor immunity requires further investigation.

## NECROPTOSIS IN CANCER THERAPY

Necroptosis may be linked to not only tumorigenesis and anti-tumor immunity, but also to the success of cancer treatments. Characterization of the precise mechanisms underlying necroptosis and the molecular switches between apoptosis and necroptosis, may have major therapeutic implications [130–131]. In particular, we will focus on converging and diverging evidence implicating necroptosis in cancer therapies, and hope that research into necroptosis will provide new targets for effective therapies.

### Necroptosis as a cancer therapeutic target

As discussed above, necroptosis is often impaired during tumorigenesis, but can be engaged by targeted pharmacological approaches. It is also clear that necroptosis plays a pivotal role in the pathogenesis of cancer. Therefore, characterization of the signal transduction pathways underlying necroptosis may highlight therapeutic targets.

#### Compounds and anticancer drugs

A growing list of compounds and anticancer drugs with various primary mechanisms of action, have been reported to initiate programmed necrosis or necroptosis in different cancer cells *via* mediated RIPK1, RIPK3, MLKL, and HMGB1 [132–166]. However, most studies on cancer and necroptosis are based on *in vitro* experiments, thus the efficacy and safety of these compounds and anticancer drugs still need investigation *in vivo* (Supplementary File 1).

#### Kinase inhibitors

Necroptosis has been implicated in the anti-tumor activity of several kinase inhibitors. Bl2536, a small molecule inhibitor of the mitotic kinase pololike kinase 1 (Plk1), has been reported to induce necroptosis in androgen-resistant prostate cancer cells [167]. Compound C, a widely used 5′-AMP-activated protein kinase inhibitor was described to induce glioma cell death through several mechanisms including necroptosis [168]. A cell permeant urokinase plasminogen activator system inhibitor 5′-benzylglycinyl-amiloride (UCD38B) also induces cell cycle independent and caspase-independent death of necroptotic glioma cells and breast cancer cells *via* apoptosis-inducing factors, independent of poly (ADP-ribose) polymerase and H_2_AX activation [169–170]. The formation of ATG5 deficient autophagosomes in response to sorafenib promotes interaction of p62 with RIPK, leading to cell death by necroptosis [171–172]. In addition, the combination of Givinostat and Sorafenib caused sustained production of ROS and activation of necroptotic relapsed/refractory Hodgkin's lymphoma cell death [172].

#### Radiation therapy and chemotherapy

In addition to the drugs and kinase inhibitors that promote necroptosis, radiation and/or chemotherapy can also induce necroptosis of cell death [173]. In tumor cells of epithelial origin, radiotherapy induces limited apoptosis, but when combined with hyperthermia has been reported to stimulate necroptosis [174–175]. Furthermore, active caspase-8 induces apoptosis in response to low-doses radiation and inhibits necrosis by cleaving RIPK1. When activation of caspase-8 was reduced by high doses radiation, the RIPK1/RIPK3 necrosome II was formed. These complexes induce necroptosis through the caspase-3-independent pathway, mediated by calpain, cathepsin B/D, and apoptosis-inducing factor (AIF) [176]. MyD88 may determine whether UV irradiation causes apoptosis or necroptosis [177]. In addition, pan-caspase inhibitors can sensitize resistant colon cancer cells to 5-FU-induced necroptosis [178]. As radiation equipment and techniques, and chemotherapies improvement, further studies will be required to explore the potential for anticancer strategies to manipulate necroptosis with minimal side effects.

#### Others

MicroRNAs (miRNAs) are highly conserved, small noncoding RNA molecules that function to regulate a wide variety of cellular processes including cell proliferation and differentiation, fate determination, signal transduction, organ development, host-viral interactions, tumorigenesis and tumor progression [179–180]. While the mechanisms by which miRNA regulate necroptotic cell death are not well understood, miRNA-874 was reported to enhance necroptosis by targeting caspase-8, which acts as a key modulator of the transition between apoptosis and necroptosis [181]. However, how miRNAs regulate other key necroptotic factors, including RIPK3, MLKL, and PGAM5, remains to be determined. In addition, nanosecond pulsed electric fields and gemcitabine induce similar cell death pathways, particularly in breast cancer MCF-7 cells; however, the two agents exhibit different mechanisms of necrosis, most likely necroptosis, in breast cancer MDA-MB-231 cells [182]. Endoplasmic reticulum stress can also trigger RIPK1 kinase dependent necroptosis [183], and viruses can induce necroptosis in ovarian carcinoma cells and neuroblastoma cell lines [184–185].

### Crosstalk between necroptosis and other forms of cancer cell death forms

Despite involving morphologically different and distinct molecular pathways, the pathways controlling different cell death modes involve complex interaction and crosstalk [186].

#### Induction of the mixed forms of death

Several studies have demonstrated that some drugs and kinase inhibitors, and heat stress, induce simultaneous apoptosis, necroptosis and autophagy in cancer cells [187–214] (Supplementary File 2). Furthermore, the combination of necroptotic inducers and standard treatments enhanced this form of cancer cell death. For example, gambogenic acid, one of the main components of Gamboge, and 5-Fu have synergistic effects on A549 cells, activating cell death through apoptotic and necroptotic mechanisms *via* the ROS-mitochondrial pathway [215]. Treatment of colorectal tumor cells with RT and hyperthermia also activates apoptosis and necroptosis [174]. These forms of cancer cell death highlight the interconnected nature of cell the involved pathways, and interdependence of the different modes of cancer cell death [22, 216].

#### Induction of apoptosis engages necroptosis

Some agents that are well-known inducers of apoptosis can also induce necroptosis under certain conditions. For example, staurosporine has been reported to trigger necroptosis in leukemia cells, and necroptosis was blocked by the RIPK1 inhibitor Nec-1 and MLKL inhibitor NSA [217]. The death receptor ligand TRAIL can also trigger necroptosis in colon and liver cancer cells at acidic extracellular PH [218–220]. Cancer cells shift from apoptosis to programmed necrosis or necroptosis when energy stores are depleted, such as during DNA repair by poly-ADP-ribose polymerase (PARP) activation, or switching of the extracellular PH in human HT29 and HepG2 cells [220–221]. However, Sosna *et al*. [222] have shown that necroptosis and PARP-1-mediated necrotic cell death follow two distinct routes to regulate necrosis. In addition, small molecule inhibitors that antagonize inhibitors of apoptotic proteins, such as second mitochondrial activator of caspases (smac) mimetics, were shown to cause necroptotic cell death in fibrosarcoma cells, breast cancer cells, and acute lymphoblastic leukemia, underscoring the potential clinical relevance of this therapeutic strategy [223–224], which is regulated by ROS [225]. Therefore, whether cancer cells undergo apoptosis or necroptosis following exposure to smac mimetics will be crucial for the implementation of therapy, and should be addressed in future studies.

#### Induction of necroptosis engages apoptosis

In contrast to the above-mentioned results, some agents inducing of necroptosis were also found to induce apoptosis under certain conditions. For example, shikomin and its analogues induced necroptosis, but in the presence of Nec-1, cells revert to apoptosis. The death mode switch is at least partially due to the conversion from mitochondrial inner membrane permeability to mitochondrial outer membrane permeability, which is associated with Bax translocation [226–227]. In addition, shikonin at low doses induces caspase dependent apoptosis, but at high doses induces necroptosis. Gene expression profiling revealed that 353 genes were significantly regulated by low dose shikonin and 85 genes by high dose shikonin. Among these genes, the transcription factor 3 and DNA damage inducible transcript 3 were highly expressed following low dose treatment, while high doses induced tumor necrosis factor expression. These findings provide novel information about the molecular mechanism of shikonin-induced apoptosis and necroptosis [228]. In combination with a previous report [16], these findings seem to support the notion that apoptosis and necroptosis may function as reciprocal backup mechanisms. Although the precise molecular mechanisms by which these agents induce cell death modes remain to be elucidated, these results indicate multiple pathways for the treatment of cancer.

#### Induction of autophagy engages necroptosis

Little is known about how autophagy is intertwined with necroptosis. However, some of the first evidence to show that autophagy could promote cell death came from a system that has gone on to become the best understood necroptosis pathway [229–232]. In this case, autophagy was shown to modulate necroptosis by selectively degrading the ROS scavenger enzyme catalase [233]. In another example, one piece of evidence suggests that autophagosomal membranes act as platforms for necrosome assembly, and serves as key sites to mediate necroptosis. For example, autophagosomes can act as key mediators of efficient necrosome formation, resulting in necroptotic cell death by Obatoclax (GX15-070), which is a compound that antagonizes Bcl-2 family proteins [152]. On the contrary, Bray *et al*. provided another example of the coordination between necroptosis and autophagy [234]. They found that concurrent mTOR inhibition by CCI-779 and inhibition of autophagosome maturation with chloroquine caused accumulation of autophagosomes that induced RIPK3-dependent and ROS-dependent necroptosis in renal cell carcinoma lines. RIPK1 may also degrade by autophagy. Overall, these data support the notion that autophagy can influence the fate of cells treated with compounds that induce necroptosis. However, the molecular underpinnings of this relationship remain largely elusive and somewhat controversial. Autophagy has been shown to induce [152, 229–233], suppress [117, 235–236] or not be involved in necroptosis [237]. Further work will be required to uncover the mechanistic ties, and to determine how these processes are controlled.

### Necroptosis overcomes therapy resistance of cancer

Cancer resistance is a major obstacle limiting the efficacy of cancer therapy. Many lines of clinical and experimental evidence have demonstrated that an apoptotic defect is the most frequent cause of cancer resistance [23]. First, since the neoplastic process is driven by oncogenic mutations that increase tumor cell number *via* activating the cell cycle and/or inhibiting the normal apoptotic process, cancer cells are genetically predisposed to apoptotic resistance. Second, since conventional anticancer therapies, regardless of their targets and mechanisms, mostly induce apoptosis, therapy resistance *via* anti-apoptotic progression appears to be inevitable, and the clones with greater selective advantages against apoptosis are destined to dominate the total cancerous cell population, forming cancers in an ever more relentless cycle [238–239].

Apoptotic defects in cancers include over-expression of anti-apoptotic proteins, mutations of pro-apoptotic proteins and the loss of caspases [239]. The blockage of a single pathway does not entirely block cell death; however, it may provoke the cell to choose an alternative death pathway [240]. Therefore, targeting the “weak point” of cancers, especially apoptotic/drug resistant cancers, is critical for the success of cancer therapy. The necroptotic pathway might be an intrinsic “weak point” under these circumstances, and thus exploitation of this alternative cell death pathway may help bypass the blockade to destroy therapy-resistant cancers [241].

Generally speaking, apoptosis can overshadow necroptosis, implying that apoptotic death is the first choice in most settings and that necroptosis acts only as an alternative to ensure cell demise [242]. However, induction of necroptosis by a small molecular compound can circumvent cancer resistance. For example, Cisplatin-based chemotherapy is currently the standard treatment for locally advanced esophageal cancer, but cancer cells have evolved several strategies to evade apoptosis. Xu *et al*. [243] indicated that RIPK3 and the autocrine production of TNF-α contribute to cisplatin sensitivity by initiating necrosis when the apoptotic pathway is suppressed or absent in esophageal cancer cells. These findings provide new insight into the molecular mechanisms underlying cisplatin-induced necroptosis, and suggest that RIPK3 is a potential marker of cisplatin sensitivity in apoptosis-resistant and advanced esophageal cancer. The inhibition of glycogen synthase kinase 3 alpha (GSK3A) or glycogen synthase kinase 3 beta (GSK3B), whose silencing bypasses drug resistance due to the loss of p53, can abolish cell viability and cloning growth *via* necroptosis. Targeting GSK3B with/without CT may thus represent a novel strategy for the treatment of chemotherapy resistant tumors [244–246]. Meanwhile, a diphtheria based fusion toxin or smac mimetric, in addition to demethylating agents, synergistically trigger cell death in cancer cells and overcome apoptosis resistance by inducing necroptosis [247–249]. In addition, Trastuzumab, a humanized recombination monoclonal antibody that binds to the extracellular domain of Her2, can simultaneously induce necroptosis and overcome the resistance of tumor cells to antibody treatment [250]. These findings raise the possibility that induction of necroptosis may be considered an alternative choice for therapeutic resistant cancers.

### Development of necroptotic inhibitors

Traditional chemotherapeutic agents are not efficient inducers of necroptosis, and more potent necroptotic pathway-specific drugs will be required to fully harness the power of necroptosis in anticancer therapy. We have reviewed the published literature on small molecule inhibition of necroptosis, and the development of specific necroptotic inducers, in combination with conventional therapeutic styles such as RT or CT, may be required to effectively treat cancers [251–252].

#### Nec-1

Small molecule compounds that inhibit programmed necrosis are not only effective tools for the study of necrotic cell death, but also have the potential to treatment for necroptosis. Nec-1 is the first necroptosis inhibitor to have been widely used *in vitro* or in animal models to study necroptosis. Nec-1 specifically inhibits necroptosis, but does not affect apoptosis and autophagy, and does not affect the general physiology of healthy cells, ATP levels, mitochondria membrane potential, plasma membrane integrity, cell shape and size, cell cycle distribution, proliferation, global mRNA expression, or intracellular ROS. The specificity of Nec-1-mediated inhibition of necroptosis has been well defined by extensive structure-activity relationship analyses, and Nec-1 was reported to allosterically inhibit the kinase activity of RIPK1, which is essential for death receptor triggered necroptosis, by interacting with its T-loop without affecting other functional domains [16]. Although many studies of necroptosis are exclusively based on experiments showing that Nec-1 inhibits cell death in the corresponding cell death model, these studies should be interpreted with caution as RIPK1 can regulate not only necroptotic but also apoptotic cell death, and therefore, Nec-1 might block apoptosis under specific conditions as well as ferroptosis except for necroptosis, in contrast to Nec-1s and ponatinib [253]. Furthermore, Linkermann *et al*. demonstrated that the *in vivo* effects of Nec-1 did not necessarily involve necrosis [254].

#### GSKs and NSA

Given that RIPK3 is involved in necroptosis, but not apoptosis, a RIPK3 inhibitor may be a more selective inhibitor of necroptosis. Necroptosis can be pharmacologically inhibited by RIPK3 inhibitors GSK-843, GSK-872, and GSK-840 [255–256]. RIPK3 silencing in cancer cells was reported to suppress the complex regulation of the apoptosis/necroptosis switch and NF-κB activation [257]. In addition, MLKL might also mediate signal transduction beyond RIPK3, as it is more widely expressed than RIPK3. It would therefore be interesting to identify the kinases with which MLKL interacts in the context of other signaling events [34]. MLKL expression, an independent prognostic biomarker in patients with early-stage resected pancreatic carcinoma, when low, is associated with a decrease in overall survival and recurrence-free survival in resected pancreatic carcinoma patients who receive adjuvant chemotherapy, even after accounting for adverse tumor characteristics and other known prognostic biomarkers [64]. Several studies reveal that NSA blocked necroptosis downstream of RIPK3 activation [29], and also targeted the N-terminal fragment of MLKL, and covalently modified MLKL through a Michael addition at a reactive amino acid residue cysteine. Therefore, NSA may represent a specific inhibitor of necroptosis. However, it should be noted that NSA does not work with murine MLKL, and currently lacks specificity.

#### Pazopanib and ponatinib

Drugs capable of inhibiting necroptosis may be useful in treating pathologies caused or aggravated by necroptotic cell death. For example, Fauster *et al*. performed a phenotypic screen for small-molecule inhibitors of TNF-alpha induced necroptosis in FADD deficient Jurkat cells using a representative panel of FDA approved drugs. Two structurally distinct kinase inhibitors, pazopanib and ponatinib, were found to be potent blockers of necroptosis, which do not protect from apoptosis [258]. Pazopanib and ponatinib abrogated phosphorylation of MLKL upon TNF-alpha induced necroptosis, indicating that both agents target a component upstream of MLKL. Importantly, ponatinib inhibited both RIPK1 and RIPK3, while pazopanib preferentially targeted RIPK1. Further studies will clarify the potential necroptosis-related clinical applications of these drugs which, given their potency in cellular assays and favorable pharmacological properties, could otherwise provide the basis for development of necroptosis inhibitors.

#### Others

In addition to Nec-1, GSKs, and NSA, a few other necroptotic inhibitors have been developed. Geserick *et al*. [259] reported that Dabrafenib, a V600E- or V600K mutated porto-oncogene serine/threonine protein kinase B-RAF, inhibited necroptosis in melanoma cells whenever RIPK3 is present. Wang *et al*. [260] reported that the histone deacetylase inhibitor vorinostat prevents TNF-α-induced necroptosis by regulating multiple signaling pathways, and suggested that histone deacetylase inhibition exerts its anti-inflammatory and cell protective effects by preventing necroptosis, an important process in inflammation and the elimination of cells. Onizawa *et al*. reported that necroptosis is inhibited by deubiquitinating enzyme A20, also known as tumor necrosis factor induced protein 3 (TNFAP3), and demonstrated that it prevents RIPK3-mediated necroptosis by blocking the ubiquitination of RIPK3 and formation of the RIPK1-RIPK3 complex [73, 261]. In addition, Carmina-Gutierrez *et al*. [262] reported that the propeptide of yeast cathepsin D inhibits necroptosis. Other necroptosis inhibitors such as Nec-7, which inhibits necroptosis independently of RIPK1, are also available [263–264].

The potential side effects triggered by necroptotic inhibitors in acute settings might differ from those of long-term anticancer treatment, and require careful evaluation. The identification of FDA approved drugs as new inhibitors of necroptosis, together with the elucidation of their mechanism of action, warrants a series of careful studies in animal models covering a wide variety of necroptosis associated pathologies. These studies may clarify the clinical potential of these necroptosis-related drugs which, given their potency in cellular assays and favorable pharmacological properties, could allow further development of clinically useful necroptosis inhibitors.

### The resistant mechanism of necroptosis

Acquired or intrinsic resistance to necroptosis may be considered a major hindrance to therapeutic success in cancers. RIPK3 expression is likely repressed during cancer development or progression, by methylation of the genomic region near its transcriptional start site. Thus RIPK3 dependent activation of MLKL and downstream-programmed necroptosis during therapeutic death is largely repressed [265]. These results suggest that RIPK3 deficient cancer patients might benefit from agents inducing RIPK3 expression prior to treatment with conventional therapeutics. Seneviratne *et al*. [266] showed that defective DNA MMR system-induced genomic instability causes the truncation of an HGF gene promoter element, which reactivates the otherwise silenced HGF gene in colonic epithelial cells. This lead to autocrine HGF production and dysregulated Met signaling in colon cancer cells, thereby promoting their resistance to necroptosis. In addition, hypoxia inducible factor-1α and glucose transporters were recently reported to colocalize at peri-necrotic regions in human colorectal tumors [267–268]. Moriwaki *et al*. [252] further reported that expression of RIPK1 and RIPK3 was suppressed by hypoxia in human colon cancer tissues. These results suggest that glucose metabolism might confer anti-necrotic resistance to hypoxia stress. Malignant cells develop adaptive mechanisms to evade necrotic death caused by the depletion of oxygen and nutrients. Huang *et al*. [269] demonstrated a novel mechanism through which glycolytic pyruvate conferred resistance to RIP-dependent necroptosis in hypoxic colorectal carcinoma *via* mitochondrial superoxide scavenging. Understanding of how cells develop resistance to hypoxic necrosis through glucose and pyruvate may aid development of novel therapeutic targets for treating colorectal carcinoma.

## FUTURE DIRECTIONS

Recently, extensive studies have elucidated the molecular mechanisms by which necroptosis is regulated and the intricate crosstalk between different cell death modes. However, our understanding of the necroptosis pathway is still at an infant stage, and the execution of necroptosis remains unclear. We still lack a biomarker for the *in vivo* detection of necroptosis. Induction of necrosis may not only eliminate tumor cells directly, but may also invoke host innate immune responses to aid clearance of tumor cells. More experimental and clinical trials will be required to clarify the potential for necroptosis-targeted cancer therapy. Although the effects of therapy-induced necroptosis in cancer remain controversial, the hypothesis provides a novel perspective and may yield a new way to overcome therapy resistance in the treatment of cancer.

## CONCLUSION

Since evasion of apoptosis represents a hallmark of human cancers, it follows that engagement of necroptosis may offer new opportunities for the development of novel treatments for apoptosis -resistant cancers. Since treatment resistance is currently the most challenging unsolved problem in oncology, therapeutic induction of necroptosis may pave an avenue for novel and more efficient treatment approaches. While a number of different anticancer compounds have been reported to trigger necroptosis in malignant cells, little is known about the precise molecular targets of these compounds. In conclusion, a better understanding of the molecular events that regulate necroptotic cell death in different types of human cancers is expected to provide exciting novel opportunities in the coming years for the therapeutic exploitation of cell death programs for the treatment of cancer. Further studies to identify novel biomarkers of necroptosis, and to develop tools for the precise characterization of necroptosis, distinct from other forms of programmed necrosis, in animal models and human disease samples, will provide crucial insight into the diagnosis, treatment, and monitoring of necroptosis-associated disease.

## SUPPLEMENTARY MATERIAL TABLES




