# Supplementary material for: Necroptosis in tumorigenesis, activation of anti-tumor immunity, and cancer therapy
Source: Oncotarget. 2016 Jul 12;7(35):57391–413. doi: 10.18632/oncotarget.10548 (PMC5302997; doi:10.18632/oncotarget.10548)
Supplement: Supplementary file 1 [file oncotarget-07-57391-s001.doc]

**Supplementary File 1:** Overview of necroptosis inducing compounds and anticancer drugs as well as their evidence

| Anticancer agents | Classification | Evidence for programmed necrosis or necroptosis | Cancer types/Lines | Reference |
| --- | --- | --- | --- | --- |
| Kuguaglycoside C | A constituent of *Momordica charantia* | Induces necroptosis through the cleaved PARP pathway as well as increases the expression and cleavage of AIF at least in part in the mechanism underlying cell necroptosis | Human neuroblastoma cells: IMR-32 | 132 |
| D-Galactose | A form of hexose | Affirm necroptosis by RIPK1 inhibitor necrostatin-1. | Malignant cells: Neuro2a, SH-AY5Y, PC-3, and HepG2 | 133 |
| Quercetin nanoliposomes | A plant extract-loaded nanoliposomal formulation | Induces programmed necrosis via the loss of mitochondrial membrane potential, cytochrome c release, ATP levels decrease, and lactate dehydrogenase activity increase. | Gioma cells: C6 | 134 |
| Azathioprine and buthionine sulfoximine | Medicine | Induces necroptosis in a mitochondrial dependent pathway including cytochrome c release and loss of membrane potential. | Hepatoma and colon carcinoma cells: HepG2, HuH7, Chang liver, LoVo, RKO, SW-48, and SW-480 | 135 |
| Izumi | Japanese black vinegar | Induces necroptosis through the RIPK3 and HMGB1 release. | Human squamous cell carcinoma cells: HSC-5 | 136 |
| Berberine | Isoquinoline alkaloid isolated from medicinal herbs | Induces programmed necrosis through mitochondrial protein Cyp-D, accompanied by ROS production and p53 translocation to mitochondrial and interaction with Cyp-D. | Prostate cancer cells: PC-3, PC-82, and LNCap | 137 |
| TRAIL | Cytokine | Induces programmed necrosis via the RIPK1 and RIPK3 increase. | Leukemia cells: U-937 and CCRF-CEM;  Gall bladder adenocarcinoma cells: Mz-ChA-1; Pancreatic adenocarcinoma cells: BxPC-3, Colo357, PancTu-I, Panc89, A818-4, and Pt45Pl;  Colorectal adenocarcinoma cells: HT-29;  Gastric adenocarcinoma cells: MKN28;  Ovary adenocarcinoma cells: SK-OV-3;  Non-small cell lung cancer cells: KNS-62;  Malignant melanoma cells: SK-Mel-28 | 138 |
| β-Lapachone | Natural compound | Induces programmed necrosis through the NQO1-dependent ROS-mediated RIPK1/PARP/AIF-dependent pathway. | Human hepatocellular carcinoma cells: SK-Hep1 | 139 |
| Deoxynyboquinone | Agent that target the redox enzymes-NQO1 | Induces programmed necrosis that consumes oxygen and generates extensive ROS. Elevated ROS levels cause extensive DNA lesions, PARP1 hyperactive, and severe NAD^+^/ATP depletion that stimulate Ca^2+^-dependent necroptosis. | Non-small cell lung cancer cells: A549, and H596;  Breast cancer cells: MCF-7, and MDA-MB-231;  Pancreatic cancer cells: MIA PaCa-2;  Prostate cancer cells: PC-3;  Sarcoma cancer cells: HT1080 | 140 |
| Diatom-derived polyunsaturated aldehydes | Teratogenic compound | Induces necroptosis via TNFR1-FADD-caspase pathway rather than RIPK. | Non-small cell lung cancer cells: A549;  Colon cancer cells: Colo 205 | 141 |
| Cobalt chloride | A reagent | Triggers necroptosis via the RIPK1/RIPK3/MLKL pathway. | Human colon cancer cells: HT-29 cell | 142 |
| Homoharringtonine | Plant extract | Induces necroptosis through the TRAIL-mediated RIPK1/RIPK3/MLKL pathway. | Gallbladder cancer cells: Mz-ChA-1;  Pancreatic carcinoma cells: Pt45P1 and A818-4 | 143 |
| Neoalbaconol | Novel small-molecular compound isolated from fungus | Induces necroptosis by regulating RIPK expression and ROS production. | Nasopharyngeal carcinoma cells: C666-1, HK1 and CNE1-LMP1 | 144 |
| 3-bromopyruvate | A glycolytic inhibitor | Induces necroptosis via a DR signaling pathway, accompanied by mitochondrial dysfunction and ROS production. | Nasopharyngeal carcinoma cells: HNE1 and CNE-2Z | 145 |
| Lycorine | A natural alkaloid | Induces programmed necrosis via RIPK1 and RIPK3 pathway, accompanied by mitochondrial dysfunction, ROS generation, ATP depletion, and DNA damage. | Multiple myeloma cells: ARH-77 | 146 |
| Dimethyl fumarate | Methyl ester of fumaric acid | Induces necroptosis through GSH depletion/ROS increase/MAPKs activation pathway. | Murine colon adenocarcinoma cells: CT26;  Human colon adenocarcinoma cells: HT29;  Human colon cancer cells: HCT116;  Human gastric cancer cells: SGC-7901 | 147 |
| Shikonin | An effective extract from *Lithospermum erythrorhixon* | Induces necroptosis via a RIPK1/RIPK3 pathway ^148^  Induces necroptosis via a RIPK1 pathway ^149^  Induces necroptosis through the RIPK1/RIPK3 pathway, accompanied by ROS production increase. ^150^  Induces necroptosis through six characteristics including morphological enzymological, and function.^151^ | Murine osteosarcoma cells: K7, K12 and K7M3;  Human osteosarcoma cells: U2OS and 143B ^148^  Rat glioma cells: C6 and human glioma cells: U87 ^149^  Breast cancer cells: MDA-MB-468 ^150^  Breast cancer cells: MCF-7 and human embryonic kidney cells: HEK293 ^151^ | 148-151 |
| Obatoclax (GX15-070) | A small-molecule inhibitor of antiapoptotic Bcl-2 protein | Triggers necroptosis by a RIPK1/RIPK3-dependent manner. ^152^  Induces necroptosis dependent on expression of RIPK1 and CYLD. ^153^ | Rhabdomyosarcoma cell line ^152^  Acute lymphoblastic leukemia cells: CME-C7-14 and CEM-C1-15. ^153^ | 152-153 |
| Polyphenon E | A green tea extract | Triggers necroptosis through a caspase-independent pathway, requiring AIF activation and PARP-1 cooperation. | Prostate cancer cells: PNT1a, PC3, LNCaP and DU145 | 154 |
| Deoxypodophyllotoxin | A naturally occurring microtubule destabilizer | Induces necroptosis through six characteristics including morphological, enzymological, and function. | Non-small cell lung cancer cells: NCI-H460 | 155 |
| Methy1 methanesulfonate | A DNA-damaging alkylating agent | Induces necroptosis through the PIG-3-ROS pathway, accompanied by the increased expression of HMGB1 and RIPK. | Lung adenoma cells: A549 | 156 |
| Green tea polyphenol | A green tea extract | Triggers necroptosis related to the translocation of Bax and Bak to mitochodria, release of cytochrome c, and activation of caspases. | Human hepatocarcinoma cells: Hep3B | 157 |
| Granulysin | A member of the saposin-like protein family | Induces necroptosis via the cathepsin B release from lysosomes to attack the mitochondria to release cytochrome *c* and apoptosis-activating factor through the processing of Bid. | Human T cell leukemia cells: Jurkat cell and cervical cancer cells: Hela cell | 158 |
| Selenite | A selenium compound | Induces necroptosis through the ROS generation, perturbation of thiols homeostasis, mitochondrial dysfunction and DNA damage. | Cervical cancer cells: Hela cell | 159 |
| FTY720 | A sphingosine analogue drug | Induces necroptosis by targeting 12PP2A/SET mediated PP2A-RIPK1 pathway. | Non-small cell lung cancer cells: A549 | 160 |
| Selenosemicarbazone metal complexes | Metal complexes including Zn, Cd, and Ni | Induces necroptosis through the ROS generation | Different cancer cells: A549, MRC-5, EA.hy926, and Hela | 161 |
| PDIA6 | Protein disulfide isomerases | Induces necroptosis of DDP resistance cells through RIPK1 pathway. | Ovarian carcinoma cell: A2780 and lung adenocarcinoma cell: A549 | 162 |
| 5-ALA-PDT | A physiological heme precursor | Induces necroptosis through the RIPK1/RIPK3 pathway. | Human glioblastoma cell: LN18;  Human leukemic monocyte lymphoma cell: U937 | 163 |
| Chal-24 | A novel chalcone derivative | Induces necroptosis through the RIPK1/RIPK3 pathway. | Non-small cell lung cancer cell: A549;  Bladder cancer cell: UM-UC-3 | 164 |
| CD40L | Ligand of a transmembrane glycoprotein | Induces necroptosis by the RIPK1/RIPK3/MLKL pathway. | Ovarian invasive low-grade serous carcinomas cells: MPSC1 and VOA1312 | 165 |
| DAPE | A membrane phospholipid | Induces RIPK1-mediated necroptosis, accompanied by producing ROS, opening CypD-dependent MPT pore, disrupting mitochondrial membrane potentials, thereby reducing intracellular ATP concentrations. | Malignant pleural mesothelioma cells: NCI-H28 | 166 |

Abbreviation: AIF: apoptosis-inducing factor; PARP: Poly (ADP-ribose) poly-merase; RIPK3: Receptor-interacting protein kinase 3; HMGB1: High-mobility group protein B1; Cyp-D: Cyclophilin-D; RIPK1: Receptor-interacting protein kinase 1; NQO1: NAD(P)H: quinine oxidoreductase 1; ROS: Reactive oxygen species; AIF: Apoptosis-inducing factor; TNFR1: Tumor necrosis factor receptor 1; FADD: Fas associated death domain; MLKL: Mixed lineage kinase domain-like protein; TRAIL: TNF-related apoptosis inducing ligand; DR: Death domain receptor; GSH: Glutathione; MAPK: Mitogen-activated protein kinase; CYLD: Cylindromatosis (turban tumor syndrome); PIG-3: P53-induced gene 3; PP2A: Protein phosphatase 2A; DDP:Cisplatin; ALA: Aminolevulinic acid; PDT: Photodynamic therapy; CD40L: CD40 ligand.
