# Supplementary material for: Necroptosis in tumorigenesis, activation of anti-tumor immunity, and cancer therapy
Source: Oncotarget. 2016 Jul 12;7(35):57391–413. doi: 10.18632/oncotarget.10548 (PMC5302997; doi:10.18632/oncotarget.10548)
Supplement: Supplementary file 2 [file oncotarget-07-57391-s002.doc]

**Supplementary File 2:** Overview of the mixed death forms inducing compounds and anticancer drugs as well as its molecular mechanisms

| Anticancer agents | Classification | Type of cell death induced | The evidence of mixed death forms | Cancer types/Lines | Reference |
| --- | --- | --- | --- | --- | --- |
| Obatoclax | A small molecule inhibitor of antiapoptotic Bcl-2 proteins | Autophagy, apoptosis, necroptosis | Induces multiple cell death with related gene expression and morphologic evidence. | MLL-AF4 ALL cells: RS4: 11 and SEM-K2 | 187 |
| Estrogen receptor β (ER-β) | Hormone receptor | Autophagy and necroptosis | Induces both cell death through the binding of the Sp1 on the PTEN promoter gene. | Human seminoma cell: TCAM2 | 188 |
| Diphtheria toxin GM-CSF | A novel fusion toxin protein | Apoptosis, necroptosis | Induces both cell death depending on the ability of the targeted toxin to inhibit protein synthesis. | Human acute myeloid leukemia cell: U937, Kusami and HL60 | 189 |
| Aur-A and Plk1 | Mitotic kinases | Apoptotic and necroptosis | Induces both cell death with phosphorylation of FADD, resulting in activation of RIPK1. | Cervical adenocarcinoma cells: Hela;  Normal lung epithelial cells: L132;  Gastric adenocarcinoma cells: AGS;  Colorectal adenocarcinoma cells: HCT15;  Pancreatic adenocarcinoma cells: CFPAC-1 | 190 |
| Sanguilutine | Natural product | Autophagy and necroptosis | Induces autophagy via GFP-LC3 puncta formation and LC3-II accumulation and affirm necroptosis with RIPK1 inhibitor necrostatin-1. | Human melanoma cells: A-375 and Mel-JuSo | 191 |
| FTY720 | A potent immunosuppressant | Autophagy, apoptosis, and necroptosis | Induces multiple cell death via RIPK1/RIPK3-ROS/JNK/p53-PI3K/AKT/mTOR/P70S6K pathway. | Human glioblastoma cells: U251MG, U87MG and A172 | 192 |
| ZD55-IFN-β | Interferon-β-armed oncolytic adenovirus | Apoptosis and necroptosis | Induces apoptosis through the cledvage of procaspase-9 and procaspase-3, as well as induces necroptosis via the MMP loss, ATP levels drop, and ROS production. | Human hepatoma cells: SMMC-7721 and Hep-3B;  Breast adenocarcinoma cells: MCF-7 and MDA-MB-231;  Embryonic kidney cells: HEK293 | 193 |
| G-TTP | HSP90 inhibitor | Autophagy, apoptosis, and necroptosis | Induces apoptosis through caspase, autophagy through Beclin-1, as well as caspase-dependent apoptosis and necroptosis via RIPK1. | Hepatocellular carcinoma cells: Hep3B;  Suspended leukemia cells: U937 | 194 |
| Sorafenib | A multikinase inhibitor | Apoptosis and necroptosis | Induces caspase-dependent apoptosis and necroptosis under conditions of inefficient caspase activation. | Myeloma cells: MM.1S, U266, H929, RPMI8226 and OPM-2 | 195 |
| Neoalbaconol | A novel small molecular compound isolated from fungus | Autophagy, apoptosis, and necroptosis | Induces multiple cell death via PDK1/PI3K/Akt signaling pathway, accompanied by consumption of glucose, ATP generation, upregulation of LC3, presence of apoptotic and necrotic morphology, RIPK1/RIPK3 colocalization and interaction, as well as rescued by 3-MA, ZVAD-fmk, and necrostatin-1. | Nasopharyngeal carcinoma cells: C666-1 and HK-1 | 196 |
| Heat stress | Physics stimuli | Apoptosis, autophagy and necroptosis | Induces apoptosis through caspase 3/7 and necroptosis rescued by necrostatin-1. ^197^  Induces apoptosis and necroptosis through the up-regulation of LC3-II and RIPK3, respectively. ^198^ | Rat hepatocytes: Clone9 ^197^  Rat hepatocellular carcinoma cells: N1S1 and As30D ^198^ | 197  198 |
| Troglitazone | A synthetic agent targeting PPARγ | Autophagy, apoptosis, and necroptosis | Induces multiple cell death with morphologic evidence, and induces apoptosis through AMPK, autophagy through LC3-II, and affirm necroptosis with RIPK1 inhibitor necrostatin-1. | Bladder cancer cells: T24 | 199 |
| 24S-OHC | Enzymatical production | Apoptosis and necroptosis | Induced necroptosis is dependent on activity of acyl-CoA: ACAT1 in SH-SY5Y cell, and induces both cell death dependent on activity of ACAT1 and caspase in Jurkat T cell. | Human neuroblastoma cells: SH-SY5Y;  Human T cell leukemia cells: Jurkat | 200 |
| SMAC-armed vaccinia virus | A tumor-targeted vaccinia virus carrying *SMAC/DIABLO* gene | Apoptosis and necroptosis | Triggers cell death via caspase-dependent apoptosis and RIPK1-dependent necroptosis with depletion of IAPs and ripoptosome assemblely. | Human hepatocellular carcinoma cells: SMMC-7721, BEL-7404 and Huh-7 | 201 |
| INF-γ and chemotherapy | Interferon combination 5-Fu or DDP | Apoptosis and necroptosis | INF-γ sensitizes chemotherapy-induced apoptosis and necroptosis through up-regulation of Egr-1. | Human head and neck squamous carcinoma cell: SCC-25 | 202 |
| Edelfosine | The prototype molecule of a family of unnatural lipids | Apoptosis and necroptosis | Induces RIPK1/RIPK3/MLKL-dependent necroptosis and triggers caspase-dependent apoptosis when low procaspase-8, RIPK1, and RIPK3. | Glioblastoma cells: U-118 MG | 203 |
| Shikonin | An effective extract from *Lithospermum erythrorhixon* | Apoptosis and necroptosis | Induces necroptosis via ROS stimulation, and induces caspase-dependent apoptosis when the presence of RIPK1 inhibitor necrostatin-1. ^204^  High dose induces necroptosis and low dose induces apoptosis. ^205^ | Breast cancer cell: T-47D ^204^  Human myeloma cells: KMS-12-PE, KMS-12-BM, RPMI-8226, KMM1, U226, KMS11, and KMS11/BTZ ^205^ | 204  205 |
| HUHS1015 | The naftopidil analogue | Apoptosis and necroptosis | Induces caspase-independent apoptosis and necroptosis in association with accumulation of AIF/ AMID in the nucleus. | Human gastric cancer cells: MKN28 | 206 |
| NPe6-PDT | Photodynamic therapy using the photosensitizer talaporfin sodium | Autophagy and necroptosis | Low dose induces autophagy with LC3-II accompanying autophagosome formation, and high dose induces necroptosis via RIPK1/RIPK3/MLKL pathway with LDH leakage. | Human glioblastoma cells: T98G | 207 |
| Eupomatenoid-5 | A neolignan isolated from *Piper regnellii* leaves | Apoptosis and necroptosis | Induces both cell death through oxidative stress, involving ROS generation, time-dependent loss of mitochondrial membrane potential, and a sudden decrease of MMP seeming to be involved to necroptosis pathway. | Human breast tumor cell: MCF-7  Human kidney tumor cell: 786-0 | 208 |
| Sesquiterpene lactone polymatin B | The extract isolated from the leaves of *Smcllanthus* | Apoptosis and necroptosis | Induces apoptosis in CCRF-CEM cells, necroptosis in CEM-ADR5000 cells through induction of RIPK1, neither apoptosis nor necroptosis in MIA-PaCa-2 cells. | T-cell cute lymphoblastic leukemia cell: CCRF-CEM; Doxorubicinresistance T-cell leukemia cell: CEM-ADR5000;  Pancreatic carcinoma cell: MIA-PaCa-2 | 209 |
| CD30 | A member of the tumor necrosis factor receptor superfamily | Apoptosis and necroptosis | RIPK1 is a regulator of CD30-mediated cell death that bears features of both, apoptosis and necroptosis. | Anaplastic large-cell lymphoma cells: DEL, SUPM2, SU-DHL1, SR 786 and Karpas 299 | 210 |
| Smac mimetic BV6 | A bivalent IAP antagonist compound | Apoptosis and necroptosis | Primes apoptosis-resistant cells lacking FADD or caspase-8 to TNF-induced, RIPK1-dependent and caspase-independent necroptosis, whereas it sensitizes apoptosis-proficient cells to TNF-mediated, caspase-dependent apoptosis. | Human WT Jurkat T-ALL, FADD-deficient, caspase-8-deficient, or caspase-8-deficient and Bcl-2-overexpressing variants of human Jurkat clones deficient in FADD, caspase-8 or caspase-8-deficient, and Bcl-2-overexpressing cells | 211 |
| Caediac glycoside (UNBS1450) | A natural and semisynthetic compound extracted from the flowering plant | Autophagy, apoptosis, and necroptosis | Induces multiple cell death through the key mechanism of mitophagy. | Neuroblastoma cells: SH-SY5Y and SK-NAS | 212 |
| OSW-1 | Molecular compound of cholestane saponin family | Apoptosis and necroptosis | Induces apoptosis and necroptosis through the mitochondrial pathway. | Human hepatocarcinoma cells: Hep3B | 213 |
| HUHS1015 | Naftopidil analogue | Apoptosis and necroptosis | Increase nuclear localization of AIF-homologous mitochondrion associated inducer of death. | Human gastric cancer cells: MKN28 and MKN45 | 214 |

Abbreviation: RIPK1: Receptor-interacting protein kinase 1; RIPK3: Receptor-interacting protein kinase 3; PTEN: Phosphatase and tensin homolog deleted from chromosome 10; Aur-A: Aurora-A; Plk1: Polo-like kinase 1; ROS: Reactive oxygen species; JNK: c-Jun N-terminal kinase; PI3K: Phosphatidylinositide 3-kinase; AKT: Protein kinase B; mTOR: Mammalian target of rapamycin; G-TPP: Gamitrinib variant containing triphenylphosphonium; PDK1: 3-phosphoinositide-dependent protein kinase 1; AMPK: Adenosine monophosphate-dependent protein kinase; ACAT1: Acyl-CoA: cholesterol acyltransferase 1; 5-Fu: 5-fluorouracil; DDP: Cisplatin; AIF: Apoptosis-inducing factor; AMID: Mitochondrion-associated inducer of death; LDH: Leakage of lactate dehydrogenase; FADD: Fas associated death domain; TNF: Tumor necrosis factor; AIF: apoptosis-inducing factor.
